# An age-based analysis of nonmedical prescription opioid use among people who use illegal drugs in Vancouver, Canada

**DOI:** 10.1186/s13011-018-0180-3

**Published:** 2018-11-27

**Authors:** Tessa Cheng, Will Small, Huiru Dong, Ekaterina Nosova, Kanna Hayashi, Kora DeBeck

**Affiliations:** 1 Faculty of Health Sciences, Simon Fraser University, Blusson Hall, Room 11300, 8888 University Drive, Burnaby, BC V5A 1S6 Canada; 2 British Columbia Centre on Substance Use, Providence Health Care, 400-1045 Howe St, Vancouver, BC V6Z 2A9 Canada; 3 School of Population and Public Health, University of British Columbia, 2206 East Mall, Vancouver, BC V6T 1Z3 Canada; 4 Centre for Applied Research in Mental Health and Addiction, SFU Faculty of Health Sciences, 515 W. Hastings Street, Vancouver, BC V6B 5K3 Canada; 5 School of Public Policy, Simon Fraser University, 515 West Hastings Street, Suite 3271, Vancouver, BC V6B 5K3 Canada

**Keywords:** Prescription opioids, Risk behavior, Addictions, Youth, Overdose

## Abstract

**Background:**

Nonmedical prescription opioid use (NMPOU) is a serious public health problem in North America. At a population-level, previous research has identified differences in the prevalence and correlates of NMPOU among younger versus older age groups; however, less is known about age-related differences in NMPOU among people who use illegal drugs.

**Methods:**

Data were collected between 2013 and 2015 from two linked prospective cohort studies in Vancouver, Canada: the At-Risk Youth Study (ARYS) and the Vancouver Injection Drug Users Study (VIDUS). Factors independently associated with NMPOU among younger (ARYS) and older (VIDUS) participants were examined separately using bivariate and multivariate generalized estimating equations.

**Results:**

A total of 1162 participants were included. Among 405 eligible younger participants (Median age = 25; Inter-Quartile Range [IQR]: 22–28), 40% (*n* = 160) reported engaging in NMPOU at baseline; among 757 older participants (Median age = 48, IQR: 40–55), 35% (*n* = 262) reported engaging in NMPOU at baseline. In separate multivariate analyses of younger and older participants, NMPOU was positively and independently associated with heroin use (younger: Adjusted Odds Ratio [AOR] = 3.12, 95% Confidence Interval [CI]: 2.08–4.68; older: AOR = 2.79, 95% CI: 2.08–3.74), drug dealing (younger: AOR = 2.22, 95% CI: 1.58–3.13; older: AOR = 1.87, 95% CI: 1.40–2.49), and difficulty accessing services (younger: AOR = 1.47, 95% CI: 1.04–2.09; older: AOR = 1.74, 95% CI: 1.32–2.29). Among the youth cohort only, NMPOU was associated with younger age (AOR = 1.12, 95% CI: 1.05–1.19), crack use (AOR = 1.56, 95% CI: 1.06–2.30), and binge drug use (AOR = 1.41, 95% CI: 1.00–1.97); older participants who engaged in NMPOU were more likely to report crystal methamphetamine use (AOR = 1.97, 95% CI: 1.46–2.66), non-fatal overdose (AOR = 1.76, 95% CI: 1.20–2.60) and sex work (AOR = 1.49, 95% CI: 1.00–2.22).

**Discussion:**

The prevalence of NMPOU is similar among younger and older people who use drugs, and independently associated with markers of vulnerability among both age groups. Adults who engage in NMPOU are at risk for non-fatal overdose, which highlights the need for youth and adult-specific strategies to address NMPOU that include better access to health and social services, as well as a range of addiction treatment options for opioid use. Findings also underscore the importance of improving pain treatment strategies tailored for PWUD.

## Background

Rates of medical and nonmedical prescription opioid use (NMPOU) are rising across Canada and the United States [1], along with shifts towards increases in the global consumption of opioids [2]. Research consistently indicates that adolescents and young adults are more likely to engage in NMPOU than older youth and adults [3–11], and evidence suggests that these differences in prescription opioid (PO) use are associated with statistically significant birth cohort effects; more recent birth cohorts have higher lifetime and past-year prevalence of prescription opioid-use disorder due to NMPOU [12]. Adolescents and young adults in the United States are more likely to initiate NMPOU than older age groups [3, 13–15], and a study of the American general population found that the most frequently reported age of NMPOU initiation was 16–18 years [16]. This effect has been attributed to the increased availability of POs [4], and other research among street-involved youth has found that the easy availability of POs facilitates NMPOU [17]. In addition, those who engage in NMPOU are more likely to be younger than those engaging in other illegal drug use [18], and adolescents and young adults are also more likely to share and receive any class of prescription medications, including POs, than older age groups [19]. For the purposes of this study, “youth”, “young adult”, and “younger age groups” are used to describe individuals in their late teens and up to the late 20’s [20]. “Adult” and “older age groups” include those over the age of majority but focuses on those in their mid-life and older.

Youth-specific strategies to address substance use are often prioritized due to the developmental harms associated with licit and illegal substances [21]; however, the prevalence of NMPOU among adults up to age 64 is significantly higher than among adults over the age of 65 [22], and the ubiquity of PO use has resulted in a significant risk of engaging in NMPOU that increases with age [12]. The increase in PO use among older individuals is especially problematic given that age-related physiological changes (e.g., drug absorption) increase the harms of PO use among older adults, such as hyperalgesia [23]. Research has also identified a key difference related to NMPOU among younger and older age groups, where pain is a more frequent motivator for engaging in NMPOU among adults [24] and youth are more likely to engage in NMPOU for its euphoric effects [6].

Although younger and older age groups are both at risk for engaging in NMPOU, the majority of research findings related to NMPOU rely on population-level sampling and national surveys that often exclude marginalized groups who are unstably housed or have low incomes. Despite previous research investigating NMPOU among people who use illegal drugs (PWUD) in Canadian and American settings [17, 25–34], there have been few if any studies characterizing age-based differences associated with NMPOU among PWUD. Given that this population already experiences numerous risks and harms related to substance use [35], this study investigates age-based differences associated with NMPOU among younger and older PWUD in Vancouver.

## Methods

Data from this study were drawn from two prospective open cohort studies: the At-Risk Youth Study (ARYS) and the Vancouver Injection Drug Users Study (VIDUS). ARYS and VIDUS use a harmonized study questionnaire and study participants can attend a study visit at either study office regardless of their cohort enrollment. ARYS and VIDUS have both been described in detail previously [36, 37]. VIDUS is a cohort of HIV-negative adult PWUD who injected illegal drugs at least once in the month prior to enrolment. Participants in the VIDUS cohort are recruited through self-referral and street outreach, which has been ongoing since 1996. In brief, ARYS has been operating since 2005 as a cohort study of street-involved youth. To be eligible, participants must be aged 14–26 years at recruitment and also have used illegal drugs other than cannabis in the past 30 days, provide written informed consent, and be “street-involved”. In this cohort, “street-involved” is defined as being absolutely, periodically, or temporarily homeless (e.g., having no fixed address, sleeping on the street, couch surfing, or staying in a shelter or hostel), and includes those who are not homeless but have used services designated for street-youth (e.g., youth-specific drop-in centres, social services, and harm reduction services) in the last year. Youths’ street involvement and eligibility to participate are assessed during a semi-structured in-person interview with an ARYS staff member.

At enrolment, and on a semi-annual basis, participants in ARYS and VIDUS complete a questionnaire that is administered by trained study staff. The questionnaire includes questions related to demographic information and drug use patterns. At each study visit, participants are provided with a stipend ($30 CDN) for their time. The ARYS and VIDUS studies have been approved by the University of British Columbia/Providence Health Care Research Ethics Board.

All ARYS and VIDUS participants who completed a study visit between December 2013 and May 2015 were eligible for the present analyses, as PO-related questions were added to the study instrument during the summer of 2013. The dependent variable for these analyses was engaging in NMPOU through any route of administration, based on responses to the question: “In the last 6 months, when you were using, which of the following non-injection prescription opiates did you use when they were not prescribed for you or that you took only for the experience or feeling they caused, and how often did you use them?” and “In the last 6 months, have you injected any of the following prescription opiates?” (yes vs. no). To identify factors associated with engaging in NMPOU, we considered a number of potential explanatory variables of interest. The following socio-demographic variables of interest were included: age (per year younger); sex; Caucasian ancestry; and homelessness, defined as having no fixed address, sleeping on the street, couch surfing, or staying in a shelter or hostel in the last 6 months. The following variables related to drug use were also included: any injection or non-injection heroin use; any injection or non-injection crack cocaine use; any injection or non-injection cocaine use; any injection or non-injection crystal methamphetamine use; binge drug use, defined as a period of using injection or non-injection drugs more often than usual; and experiencing a non-fatal drug overdose due to injection or non-injection drug use, based on responses to the question “In the last 6 months, have you overdosed by accident (i.e., where you had a negative reaction from using too much drugs)?”. Behavioural and socio-structural risk factors hypothesized to be associated with NMPOU included: regular employment, defined as having a regular job, temporary work, or being self-employed; drug dealing, defined as selling drugs as a source of income; engaging in sex work, defined as exchanging sex for money, drugs, gifts, food, clothes, shelter or favours; incarceration, defined as being in detention, jail, or prison; and difficulty accessing services, based on responses to the question “In the last 6 months, was there a time you were in need of a service (e.g., housing, counselling) but could not obtain it?”. All variables were binary and referred to activities, behaviours, and experiences in the previous 6 months unless otherwise indicated.

All analyses were conducted separately for ARYS and VIDUS participants, using cohort enrollment as a proxy marker for younger and older age groups, respectively. At the time of enrollment, ARYS participants must be between 14 and 26 years old; however, as a prospective cohort study with substantial resources devoted to maintaining follow-up, the ARYS participant pool necessarily includes participants who are older than 26. These participants and their data are not excluded from the analyses given that this data provides a rich source for tracking and understanding long-term risk trajectories associated with street-involvement during a key developmental phase. While this practice may limit the applicability of our results to street-involved adolescents, we controlled for possible biases associated with these older participants in the youth cohort by including the continuous “per year younger” variable in the analyses to ensure that age differences within the cohort were accounted for. For consistency and to similarly control for a wide age range within the cohort, we also included the “per year younger” variable in the VIDUS analysis.

First, a descriptive analysis of the study sample was conducted using Pearson’s chi-square test. Characteristics for participants who reported nonmedical prescription opioid use (NMPOU) were measured at their first visit (during the study period: 2013–2015), which involved a report of NMPOU; characteristics for all other participants were measured from the first study visit during the study period. Second, to model factors associated with engaging in NMPOU over time and to analyse longitudinal correlated within-subject data [38, 39], generalized estimating equation (GEE) analyses were performed. These methods provide standard errors adjusted by multiple observations per person using an exchangeable working correlation structure. The GEE estimating mechanism uses all available pairs method to encompass any missing data from dropouts or other intermittent missing. All non-missing pairs of data are used in the estimators of the working correlation parameters [40].

As a first step, GEE bivariate analyses were conducted to determine factors associated with engaging in NMPOU. Variables significant in the bivariate analyses at *p* < 0.10 were considered for a full multivariate model. A backwards selection procedure was used to identify the model with the best overall fit as indicated by the lowest quasi-likelihood under the independence model criterion (QIC) value [41]. The QIC value was selected instead of the more recently developed Correlation Information Criterion (CIC), as the CIC is used to select the appropriate intracluster correlation structure, and is not used for covariate selection; we required a mechanism that could address both these needs simultaneously [42]. All statistical analyses were performed using SAS software version 9.4 (SAS, Cary, NC). All *p*-values were two sided.

## Results

A total of 405 ARYS and 757 VIDUS participants were eligible for this study. For ARYS, 313 (77.3%) participants were enrolled before December 2013 and 92 (22.7%) participants were newly enrolled during the study period. For the 313 participants, the median age at the first study visit within the study period for the current analysis was 26 (IQR: 23–28); and for the 92 participants, the median age at study enrollment was 21 (IQR: 20–23). For VIDUS, 697 (92.1%) participants were enrolled before December 2013 and 60 (7.9%) participants were newly enrolled during the study period. Among these 697 participants, the median age at the first study visit within study period was 49 (IQR: 42–55); and for the 60 newly recruited participants, the median age at study enrollment was 31 (IQR: 28–34).

The number of ARYS participants with at least one study follow-up visit was 294 (72.6%) and ARYS participants attended a median of 2 study visits (IQR: 1–3). A total of 646 (85.3%) VIDUS participants had at least one study follow-up visit and attended a median of 3 study visits (IQR: 2–3). The first ARYS observation used in this study was recorded on December 2, 2013 and the last observation was recorded on May 28, 2015. ARYS participants contributed a total of 889 observations, of which 239 (26.9%) included a report of NMPOU. The first VIDUS observation used in this study was recorded on December 2, 2013 and the last was recorded on May 29, 2015. VIDUS participants contributed 1877 observations, of which 411 (21.9%) included a report of NMPOU. Among 405 ARYS participants included in this analysis, 39.5% (*n* = 160) reported engaging in NMPOU throughout the study period. Among a total of 757 VIDUS participants, 34.6% (*n* = 262) reported engaging in NMPOU throughout the study period.

Among ARYS participants in this sample, 135 (33.3%) were female, and 250 (61.7%) were of Caucasian ethnicity; VIDUS participants were 33.9% (*n* = 257) female and 59.7% (*n* = 452) Caucasian. Descriptive statistics for younger (ARYS) and older (VIDUS) participants are displayed in Tables 1 and 2. The bivariate and multivariate analyses for younger participants are displayed in Table 3, and Table 4 displays the bivariate and multivariate analyses for older participants. In the multivariate analyses, engaging in NMPOU was independently and positively associated with the following factors among both younger and older participants: injection or non-injection heroin use (ARYS: Adjusted Odds Ratio [AOR] = 3.12, 95% Confidence Interval [CI]: 2.08–4.68; VIDUS: AOR = 2.79, 95% CI: 2.08–3.74); drug dealing (ARYS: AOR = 2.22, 95% CI: 1.58–3.13; VIDUS: AOR = 1.87, 95% CI: 1.40–2.49); and difficulty accessing services (ARYS: AOR = 1.47, 95% CI: 1.04–2.09; VIDUS: AOR = 1.74, 95% CI: 1.32–2.29).

**Table 1:** Characteristics of younger participants stratified by engaging in nonmedical prescription opioid use over the study period, 2013–2015 (*n* = 405)

Characteristic^a^	Total (%) (*n* = 405)	Nonmedical Prescription Opioid Use		*p* - value
		Yes (%) (*n* = 160)	No (%) (*n* = 245)	
Age (M [IQR])	25 (22–28)	23 (21–26)	26 (23–28)	< 0.001
Any cocaine use^b, c^	123 (30.4)	60 (37.5)	63 (25.7)	0.012
Any crack cocaine use^b, c^	138 (34.1)	70 (43.8)	68 (27.8)	< 0.001
Any crystal meth use^b, c^	268 (66.2)	121 (75.6)	147 (60.0)	0.001
Any heroin use^b, c^	193 (47.7)	114 (71.3)	79 (32.2)	< 0.001
Any non-fatal overdose^b, c^	93 (23.0)	54 (33.8)	39 (15.9)	< 0.001
Binge drug use^b, c^	208 (51.4)	108 (67.5)	100 (40.8)	< 0.001
Caucasian ancestry	250 (61.7)	100 (62.5)	150 (61.2)	0.796
Difficulty accessing services^b, d^	151 (37.3)	73 (45.6)	78 (31.8)	0.005
Drug dealing^b^	131 (32.3)	78 (48.8)	53 (21.6)	< 0.001
Female	135 (33.3)	49 (30.6)	86 (35.1)	0.350
Homeless^b^	202 (49.9)	95 (59.4)	107 (43.7)	0.002
Incarceration^b^	57 (14.1)	26 (16.3)	31 (12.7)	0.297
Regular employment^b^	191 (47.2)	81 (50.6)	110 (44.9)	0.259
Sex work^b^	48 (11.9)	26 (16.3)	22 (9.0)	0.027

a. Comparison is yes versus no unless otherwise specified

b. Refers to activities, behaviours, and experiences in the last six months

c. Includes injection and non-injection drug use

d. Includes health and social services

**Table 2:** Characteristics of older participants stratified by engaging in nonmedical prescription opioid use over the study period, 2013–2015 (*n* = 757)

Characteristic^a^	Total (%) (*n* = 757)	Nonmedical Prescription Opioid Use		*p* - value
		Yes (*%*) (*n* = 262)	No (%) (*n* = 495)	
Age (M [IQR])	48 (40–55)	47 (38–53)	49 (41–56)	< 0.001
Any cocaine use^b, c^	205 (27.1)	98 (37.4)	107 (21.6)	< 0.001
Any crack cocaine use^b, c^	332 (43.9)	135 (51.5)	197 (39.8)	0.002
Any crystal meth use^b, c^	223 (29.5)	124 (47.3)	99 (20.0)	< 0.001
Any heroin use^b, c^	362 (47.8)	200 (76.3)	162 (32.7)	< 0.001
Any non-fatal overdose^b, c^	60 (7.9)	43 (16.4)	17 (3.4)	< 0.001
Binge drug use^b, c^	235 (31.0)	118 (45.0)	117 (23.6)	< 0.001
Caucasian ancestry	452 (59.7)	164 (62.6)	288 (58.2)	0.239
Difficulty accessing services^b, d^	149 (19.7)	78 (29.8)	71 (14.3)	< 0.001
Drug dealing^b^	171 (22.6)	95 (36.3)	76 (15.4)	< 0.001
Female	257 (33.9)	92 (35.1)	165 (33.3)	0.622
Homeless^b^	149 (19.7)	76 (29.0)	73 (14.7)	< 0.001
Incarceration^b^	51 (6.7)	28 (10.7)	23 (4.6)	0.002
Regular employment^b^	202 (26.7)	59 (22.5)	143 (28.9)	0.059
Sex work^b^	72 (9.5)	36 (13.7)	36 (7.3)	0.004

a. Comparison is yes versus no unless otherwise specified

b. Refers to activities, behaviours, and experiences in the last six months

c. Includes injection and non-injection drug use

d. Includes health and social services

**Table 3:** Bivariate and multivariate GEE analyses of factors associated with engaging in nonmedical prescription opioid use among younger participants (*n* = 405)

Characteristic^a^	Unadjusted		Adjusted	
	Odds Ratio (95% CI)	*p -* value	Odds Ratio (95% CI)	*p -* value
Age (per year younger)	1.18 (1.12–1.25)	< 0.001	1.12 (1.05–1.19)	< 0.001
Any cocaine use^b, c^	1.68 (1.18–2.39)	0.004	1.31 (0.88–1.95)	0.181
Any crack use^b, c^	2.16 (1.53–3.05)	< 0.001	1.56 (1.06–2.30)	0.023
Any crystal meth use^b, c^	2.06 (1.42–2.98)	< 0.001	–	
Any heroin use^b, c^	4.82 (3.34–6.96)	< 0.001	3.12 (2.08–4.68)	< 0.001
Any non-fatal overdose^b, c^	2.24 (1.61–3.12)	< 0.001	1.43 (0.97–2.10)	0.070
Binge drug use^b, c^	2.36 (1.76–3.15)	< 0.001	1.41 (1.00–1.97)	0.049
Caucasian ancestry	1.24 (0.85–1.80)	0.269	–	
Difficulty accessing services^b, d^	1.70 (1.27–2.27)	< 0.001	1.47 (1.04–2.09)	0.030
Drug dealing^b^	2.76 (2.03–3.75)	< 0.001	2.22 (1.58–3.13)	< 0.001
Female	0.91 (0.61–1.36)	0.662	–	
Homeless^b^	1.59 (1.16–2.17)	0.004	–	
Incarceration^b^	1.32 (0.88–1.99)	0.177	–	
Regular employment^b^	1.23 (0.92–1.64)	0.168	–	
Sex work^b^	2.11 (1.35–3.29)	< 0.001	–	

a. Comparison is yes vs. no unless otherwise specified

b. Refers to behaviours, activities, and experiences in the last six months

c. Includes injection and non-injection use

d. Includes health and social services

**Table 4:** Bivariate and multivariate GEE analyses of factors associated with engaging in nonmedical prescription opioid use among older participants (*n* = 757)

Characteristic^a^	Unadjusted		Adjusted	
	Odds Ratio (95% CI)	*p -* value	Odds Ratio (95% CI)	*p -* value
Age (per year younger)	1.03 (1.10–1.04)	< 0.001	0.99 (0.97–1.00)	0.120
Any cocaine use^b, c^	1.53 (1.18–1.99)	0.001	–	
Any crack use^b, c^	1.42 (1.12–1.81)	0.004	–	
Any crystal meth use^b, c^	2.95 (2.25–3.88)	< 0.001	1.97 (1.46–2.66)	< 0.001
Any heroin use^b, c^	3.81 (2.90–4.99)	< 0.001	2.79 (2.08–3.74)	< 0.001
Any non-fatal overdose^b, c^	3.09 (2.12–4.48)	< 0.001	1.76 (1.20–2.60)	0.004
Binge drug use^b, c^	1.71 (1.37–2.14)	< 0.001	–	
Caucasian ancestry	1.20 (0.90–1.60)	0.225	–	
Difficulty accessing services^b, d^	2.11 (1.65–2.69)	< 0.001	1.74 (1.32–2.29)	< 0.001
Drug dealing^b^	2.75 (2.10–3.58)	< 0.001	1.87 (1.40–2.49)	< 0.001
Female	0.97 (0.73–1.31)	0.864	–	
Homeless^b^	2.05 (1.50–2.79)	< 0.001	–	
Incarceration^b^	2.28 (1.47–3.54)	< 0.001	1.55 (0.98–2.44)	0.061
Regular employment^b^	0.78 (0.60–1.00)	0.055	–	
Sex work^b^	2.05 (1.41–2.98)	< 0.001	1.49 (1.00–2.22)	0.049

a. Comparison is yes vs. no unless otherwise specified

b. Refers to behaviours, activities, and experiences in the last six months

c. Includes injection and non-injection use

d. Includes health and social services

Factors positively and independently associated with NMPOU among younger (ARYS) participants only included: younger age (AOR = 1.12, 95% CI: 1.05–1.19); crack cocaine use (AOR = 1.56, 95% CI: 1.06–2.30); and binge drug use (AOR = 1.41, 95% CI: 1.00–1.97). Among older (VIDUS) participants only, engaging in NMPOU was independently and positively associated with injection or non-injection crystal methamphetamine use (AOR = 1.97, 95% CI: 1.46–2.66), non-fatal overdose (AOR = 1.76, 95% CI: 1.20–2.60), and sex work (AOR = 1.49, 95% CI: 1.00–2.22).

## Discussion

Similar proportions of younger and older PWUD reported engaging in NMPOU in this study (40% of ARYS; 35% of VIDUS), and in separate multivariate analyses, NMPOU was positively associated with heroin use, drug dealing, and difficulty accessing services among both age groups. In addition, younger participants who engaged in NMPOU were more likely to be younger, use crack cocaine, and engage in binge drug use; older participants who engaged in NMPOU were more likely to use crystal methamphetamine, report a recent non-fatal overdose, and engage in sex work. While younger and older PWUD in these analyses shared risk factors for engaging in NMPOU, this study also found important differences between these two age groups that highlight opportunities to develop targeted efforts that address NMPOU and unique risks for each age group.

The association between NMPOU and age was not found to be statistically significant in the analysis of older participants, which suggests that birth cohorts were not a meaningful indicator of NMPOU among this sample. Conversely, the association between NMPOU and younger age was statistically significant among younger participants, and this finding may reflect recent trends where PO use is increasingly prevalent among young age groups [4, 12, 43]. The increasing use of POs has been attributed to the availability of POs [4, 17, 44], although the increased risk for younger birth cohorts using POs may be more strongly related to the linkages between youth observing their parents modelling substance use (i.e., PO use) and then concluding that PO use is safe [4]. Preventing initiation into injection drug use for youth who engage in NMPOU is key to reducing the sequelae of harms associated with intensifying substance use, and future research investigating prevention mechanisms is needed.

Younger and older participants who engaged in NMPOU were significantly more likely to use heroin, which previous research has found to be used as a replacement for POs when PO availability is low [45]. Although heroin and POs are both opioids and central nervous system depressants, POs are originally obtained from regulated healthcare sources, and heroin is only available through unregulated illegal drug markets. Acquiring substances from the street is especially problematic in our study setting, where the toxic synthetic opioid fentanyl has adulterated a substantial proportion of the illegal drug supply [46, 47], and fentanyl-related overdose mortality has increased alarmingly in various settings across Canada and the United States [48–51]. To reduce reliance on illegal heroin, oral medications such as buprenorphine/naloxone (Suboxone), methadone, naltrexone, and slow release morphine have been recommended in recent opioid treatment guidelines as effective treatments [52], and injectable opioids such as diacetylmorphine and hydromorphone are emerging as options for treatment-refractory opioid dependence [53, 54]. The international and North American evidence base for heroin assisted treatment (diacetylmorphine) is strong [53–55], and although less research has been conducted on hydromorphone as an injectable opioid treatment, scaling up these treatments may be an important tool to reduce NMPOU and exposure to contaminated illegal drugs in Vancouver. It is important to note that PWUD have long advocated for access to a wider spectrum of opioid agonist treatments through collaborations with researchers and coordinated advocacy efforts [56–61]. This study did not control for intentional or unintentional exposure to fentanyl or other illicitly manufactured synthetic opioids, and future research using the ARYS and VIDUS cohorts would benefit from including exposure to fentanyl in analyses.

More differences than similarities were found with illegal substance use patterns between younger and older participants. Despite both younger and older PWUD who engaged in NMPOU being significantly more likely to engage in heroin use, younger participants who engaged in NMPOU were more likely to also engage in crack cocaine use and binge drug use, while older participants were more likely to use crystal methamphetamine in addition to engaging in NMPOU. Crystal methamphetamine use may be an important marker of risk for NMPOU among older individuals in Vancouver, and this finding is concerning given local reports of increasing crystal methamphetamine use among adults in Vancouver [62–64]. Historically, crystal methamphetamine use has been more prevalent among street-involved youth in Vancouver [36], as 66% of all participants in the younger age group reported using crystal methamphetamine at baseline; in addition, crystal methamphetamine has been associated with initiating injection drug use among this cohort of youth [65].

The findings from the multivariate model indicate that NMPOU among younger participants was not associated with a significantly increased risk for non-fatal overdose, whereas older participants who engaged in NMPOU were significantly more likely to report a recent non-fatal overdose; however, it should be noted that the confidence intervals for these adjusted odds ratios overlap considerably and these findings may be attributable to differences in selection criteria for the ARYS and VIDUS cohorts. This null result for youth was unexpected given the increased rate of overdose associated with PO use [66], and that the comparison group in this analysis included non-opioid users. These findings align with previous research findings that older age is associated with mortality due to unintentional PO-related overdose [67]. Further research investigating protective factors associated with a lower risk of overdose among youth who engage in NMPOU is needed, which may include specific routes of administration or harm reducing practices related to using a substance with a known dosage and purity (e.g., prescription opioids versus unregulated heroin of unknown purity and composition). Regardless, harm reduction services are critical to ensure that older PWUD, as well as younger PWUD, who engage in NMPOU have access to overdose prevention and reversal services such as supervised drug consumption spaces/services and the widespread distribution of Naloxone/Narcan in the community and among PWUD. The reach of harm reduction services may also be increased by strategically mobilizing key peers within PWUDs’ social networks, which are an untapped resource and could become important facilitators of harm reduction supplies and information [68, 69].

Improved access to health and social services is also especially important in Vancouver, as this study found that older and younger PWUD who engage in NMPOU were more likely to report difficulty accessing services. Despite a saturation of services in the neighbourhoods where ARYS and VIDUS participants primarily live and congregate (the Downtown South and Downtown Eastside, respectively), there remain important gaps in service design and access. Previous research among youth in the ARYS cohort found that local youth-focused shelters and housing services had strict rules governing entry into- and continued use of- the service that deterred participants; conversely, adult or non-youth-specific services were perceived as unsafe or inappropriate for youth [70]. Qualitative research among the VIDUS cohort has found that a local supervised injection site often has long wait times that result in people giving up and using drugs elsewhere [71] and “area restrictions” used by police to prohibit entry into “drug scenes” impedes access to services and supports tailored for PWUD and are often specifically located in areas with high drug use [72]. This finding among younger PWUD is consistent with previous research from around the world; youth who use illegal drugs often experience difficulty accessing services due to stigma and discrimination from service providers, as well as a lack of youth-centric health and social services that are preferred but not widely available [73, 74]. Although the results indicate high rates of illegal polysubstance use in the ARYS and VIDUS cohorts, participants who only engage in NMPOU may experience additional difficulties accessing harm reduction services that are tailored to people who use illegal drugs, since they and their social networks may be outside the scope of outreach activities conducted by these services [75, 76]. More research is required to better understand specific barriers to accessing health and social services among youth and adults who engage in NMPOU, as well as inform effective solutions to fill an important service gap for these individuals.

Participants in both age groups who engage in NMPOU were more likely to report recent drug dealing, and older participants were more likely to report sex work. Drug dealing has been associated with more intense substance use among PWUD of Caucasian or white ethnicity [77], and dealing illegal drugs has been associated with a higher likelihood of NMPOU among American youth [78]. Our findings reflect the well-established relationship between socio-economic marginalization and drug dealing [40, 79], and align with consistent research findings that income from drug dealing [80] and sex work [81] is often used to sustain ongoing substance use. Despite aligning with previous research, it is not clear why drug dealing and sex work remained significantly associated with engaging in NMPOU after controlling for other illegal drug use; more research is needed investigating NMPOU and income generation among individuals not typically recruited in population-level surveys. Given that difficulty accessing services was also significantly associated with NMPOU among younger and older participants, there is a clear need to improve employment and other services for PWUD who engage in NMPOU as an intervention to increase socio-economic independence, particularly among people who engage in sex work. Similar efforts to facilitate entry into evidence-based addiction treatments for opioid use such as opioid agonist treatment, heroin assisted treatment, and injectable opioid therapy may reduce the prevalence of NMPOU and risky income generating activities.

As there is a lack of evidence to support the effectiveness of POs for treating chronic pain [82], safer prescribing to limit the supply and availability of POs is important for reducing the incidence and prevalence of NMPOU in the study setting. However, as the supply of POs becomes more restricted, close surveillance and a suite of interventions are needed to ensure that those who engage in NMPOU are not at greater risk for substituting PO use with contaminated illegal drugs that have increased overdose risks. Low barrier harm reduction services to connect with those who engage in NMPOU and have had difficulty accessing services may also be an important link to supportive healthcare services, social services, and a range of treatment options for opioid use among younger and older age groups. In addition, future research investigating the correlates of incident and recurrent NMPOU over time is needed to better understand how access to services and changes in healthcare practices impact NMPOU among high-risk individuals who use illegal drugs.

There are limitations to this research. First, ARYS and VIDUS participants may not be representative of all PWUD in Vancouver and the results therefore not generalizable to other settings in the city. However, extensive street-based and outreach efforts were undertaken in order to recruit a representative sample, and the socio-demographic characteristics of participants in the ARYS and VIDUS studies are similar to other studies in Vancouver [83, 84]. Second, this study compares data from two cohort studies with different inclusion criteria, which may result in cohort or selection effects. These cohort effects may affect the results related to homelessness and injection drug use, as these risk factors are also inclusion criteria for ARYS and VIDUS, respectively. Given the longitudinal nature of these cohort studies and extensive efforts to track participants over multiple study visits, the ARYS and VIDUS studies have observed changes in behaviours and risk factors over time. For example, we have previously reported on transitions out of homelessness among ARYS participants [85] and injection cessation among VIDUS participants [86, 87]. In addition, ARYS and VIDUS have previously been combined in quantitative and qualitative analyses [72, 88–90]. Third, social desirability and recall bias may have resulted in erroneous reporting of our outcome and independent variables. Previous research on substance use has found discrepancies between self-reported substance use and bioassay test results among American adult male arrestees [91] and noted concerns that youth may not be truthful about substance use when speaking with authority figures who are able to assign punishment [92]. Despite these findings, self-reported substance use, criminality, and HIV-related risk-taking among PWUD has also been deemed sufficiently reliable and valid [93]. Training and engaging PWUD (“peers”) in survey administration and data collection methods when conducting substance use research may be an important mechanism to reduce social desirability response biases and increase capacity within communities of PWUD [94, 95]. To reduce socially desirable reporting from participants, the ARYS and VIDUS interviewers are trained in building trust and rapport, and study instruments situate sensitive questions towards the end of the questionnaire to allow interviewers to build rapport with participants. Although some socially desirable reporting is inevitable, any such reporting from participants would be expected to under-estimate the prevalence of sensitive risk factors and therefore our findings likely represent conservative estimates. Less is known about the accuracy of self-reporting NMPOU among PWUD; however, efforts to improve the accuracy of reporting NMPOU among ARYS and VIDUS participants included using both the generic and brand name of POs, and showing pictures of a wide variety of POs during study visits. Fourth, no other information about the circumstances surrounding non-fatal overdose were included in this analysis (e.g., what substance was used at the time of overdose, whether substances were used alone or with another person, or whether fentanyl contributed to the overdose); however, the focus of this study was whether recent NMPOU was an independent marker for overdose among other risk factors. A more detailed investigation of the circumstances of overdose among people who engage in NMPOU is outside the scope of this analysis but is a promising direction for future research.

## Conclusions

The shared risk factors among younger and older participants who engage in NMPOU underscore the importance of addressing barriers to accessing health and social services, as people who engage in NMPOU appear to be particularly under-supported and under-served by existing services for PWUD. Adult PWUD who engage in NMPOU are also at greater risk of overdose, which highlights the need for youth and adult-specific strategies that focus on reducing high intensity substance use among youth and providing low barrier overdose prevention and reversal services for adults. There is an urgent need to design and implement initiatives to improve healthcare providers’ skills with managing and treating substance dependence [96], as well as developing pain treatment strategies tailored for PWUD [26]. In addition to developing services that address youths’ and adults’ unique needs, policy-makers and healthcare providers are urged to reduce systematic barriers to a range of addiction treatment options for opioid use that may contribute to reductions in the prevalence of NMPOU and provide an additional benefit of preventing other PWUD from initiating injection drug use [97].

## Abbreviations

AOR: Adjusted Odds Ratio
ARYS: At-Risk Youth Study
CI: Confidence Interval
GEE: Generalized Estimating Equation
IQR: Inter-Quartile Range
NMPOU: Non-Medical Prescription Opioid Use
PO: Prescription Opioid
PWUD: People who use Illegal Drugs
QIC: Quasi-Likelihood under the Independence model Criterion
VIDUS: Vancouver Injection Drug Users Study
